# Transcription factor KLF2 is associated with the dysfunctional status of NK cells and the prognosis of pediatric B-ALL patients

**DOI:** 10.3389/fonc.2024.1456004

**Published:** 2025-01-21

**Authors:** Fang Wu, Huimin Xu, Benshan Zhang

**Affiliations:** Department of Hematology and Oncology, The Affiliated Children’s Hospital of Xiangya School of Medicine, Central South University (Hunan Children’s Hospital), Changsha, Hunan, China

**Keywords:** NK cells, KLF2, proliferation, pediatric B-ALL, therapeutic response, prognosis

## Abstract

**Background:**

Natural killer cells, an important component of the innate immune system, can directly recognize and lyse virally infected or transformed cells. However, NK cells fail to restrain the growth of malignancies, such as B-cell acute lymphoblastic leukemia (B-ALL). The molecular genetics of NK cells in the B-ALL bone marrow microenvironment and the mechanisms underlying the inhibited function of NK cells at the single-cell level remain largely elusive.

**Methods:**

In this study, we studied the frequency and absolute number of NK cells in peripheral blood samples collected from 43 healthy volunteers and 104 pediatric B-ALL patients diagnosed at Hunan Children’s Hospital. We also analyzed published single-cell RNA sequencing (scRNAseq) data from B-ALL and normal bone marrow samples using unsupervised clustering. Our findings were further validated using bulk transcriptomic data and clinical data from a cohort of 139 B-ALL bone marrow samples.

**Results:**

We found that the frequency and number of NK cells were significantly decreased in the bone marrow and peripheral blood of B-ALL patients. In-depth analysis of scRNAseq data identified 12 NK cell clusters. Among them, the C2 cluster, which is present in healthy bone marrow but reduced in B-ALL bone marrow, displays overexpression of a transcription factor KLF2 and a significant downregulation of the “leukocyte proliferation” pathway. Furthermore, we found that the expression of KLF2 in B-ALL at diagnosis was positively correlated with the percentage of leukemia cells and the positive rate of minimal residual disease (MRD), indicating that KLF2 is a marker of poor prognosis.

**Conclusion:**

There are dramatic differences at the single-cell level in the transcriptomics of NK cells between healthy donors and B-ALL patients. A transcription factor, KLF2, which is enriched in the C2 cluster of NK cells, has been suggested to regulate the proliferation of NK cells and is associated with poor prognosis of pediatric B-ALL.

## 1 Introduction

Acute lymphoblastic leukemia (ALL) is the most common malignancy in children (1). It is estimated that 70%–75% of pediatric ALL cases are due to precursor B-cell acute lymphoblastic leukemia (B-ALL). Although cure rates for pediatric acute ALL approach 90%, outcomes for relapsed or refractory disease subgroups and salvage rates remain poor (2, 3). Since conventional chemotherapy is optimized currently to near-maximal tolerable intensity, novel approaches such as immunotherapy and adoptive transfer of immune cells are vital for improving outcomes in pediatric B-ALL.

Natural killer (NK) cells play a critical role in the innate immune response against malignancies, including leukemia (4, 5). Humans and mice that lacking NK cells have an increased incidence of cancers (6). However, the application of NK cells in treating tumors, including in hematopoietic cell transplantation (HCT), adoptive NK cell immunotherapy, and through the use of antibodies, cytokines, or drugs that augment NK cell function, has shown varying levels of success (7–9). For leukemia patients undergoing NK cell-mediated immunotherapy, the benefits are often short-lived due to challenges such as limited NK cell numbers, short lifespan, and functional exhaustion (1, 10). Previous studies indicate that NK cells become dysfunctional as B-ALL progresses, allowing malignant leukemia cells to escape immune surveillance (11, 12). Identifying the mechanisms underlying NK cell dysfunction could significantly facilitate us to reduce the relapse rate and improve the prognosis of B-ALL.

Transcriptomic analysis at the single-cell level has profoundly enhanced our understanding of the heterogeneity and immune-suppressive tumor microenvironment. With the state-of-the-art single-cell RNA sequencing (scRNAseq) technology, we can profile the bone marrow of B-ALL patients at single-cell resolution, providing greater insight into the behavior of NK cells in the B-ALL bone marrow. In this current study, we evaluated the frequency and number of NK cells isolated from the peripheral blood and bone marrow of B-ALL patients, finding a significant decrease. We then reanalyzed the NK compartment in published scRNAseq data of B-ALL bone marrow samples and found that the transcription factor Kruppel-like factor 2 (KLF2) may regulate NK cell proliferation, and is associated with poor prognosis of B-ALL.

## 2 Materials and methods

### 2.1 Blood cells and bone marrow specimen reparation

Between January 2020 and May 2023, we collected peripheral blood samples from 43 healthy volunteers and 104 patients with newly diagnosed pediatric B-ALL, while bone marrow samples were obtained from 139 B-ALL patients at the Hematology Department of Hunan Children’s Hospital. Prior to sample collection, all patients were required to provide written informed consent.

The study protocols were approved by the Ethics Committee of Hunan Children’s Hospital, Xiangya School of Medicine, Central South University. Informed consent was obtained from each patient and healthy volunteer in accordance with the Declaration of Helsinki. Sample collection and usage were carried out in strict adherence to institutional guidelines for the experimental use of human tissues.

Samples were collected in EDTA-coated tubes and immediately stored at 4°C. They were then used in flow cytometry experiments in the laboratory department. After acquisition, bone marrow specimens were promptly sent to a company for whole-gene transcriptome sequencing (RNA-seq) analysis.

### 2.2 Flow cytometry analysis of PBMC and RNA-seq analysis of bone marrow

Leukocyte and total lymphocyte analyses were performed immediately after blood sample collection using the Sysmex-XN A1 automatic hematology analyzer (Sysmex, Kobe, Japan). For lymphocyte subset analysis, 2-ml aliquots of whole blood were prepared from each participant and processed immediately to ensure homogeneous treatment. Each sample was divided, and 50-µl aliquots were incubated with a cocktail of four different fluorescein-conjugated antibodies (FITC-PE-PerCP-APC) targeting immunocytes: B cells (CD3− CD19+), NK cells (CD3− CD16+ CD56+), and T cells (CD3+). After 30 min in the dark at room temperature, erythrocytes were removed using lysis solution (BD, 349202). Samples were then centrifuged, and the supernatants were removed. Cells were washed in phosphate-buffered saline (PBS) and resuspended in PBS. Antibodies used in the study are listed in Online Resource 1. Acquisition and data analysis were then performed on a BD Accuri C6 flow cytometer (Becton Dickinson, Milan, Italy) with BD C6 software (version 1.0.264.21). To ensure high-quality and consistent results, the flow cytometry instrument was calibrated daily, and antibody fluorescence intensity was monitored weekly. Bone marrow samples were sent to the Kindstar Global Company (Wuhan, China.) for RNA-seq analysis.

### 2.3 Single-cell sequencing data

Single-cell sequencing data (GSE130116) from the bone marrow of four healthy donors and seven newly diagnosed primary B-ALL patients were downloaded from the Gene Expression Omnibus (GEO) database. These data were utilized to analyze the expression and functional differences of NK cells between healthy individuals and patients. We applied the RunTSNE function with default settings and 30 PCA dimensions for dimensionality reduction. Cell populations were identified using the FindAllMarkers function in the integrated dataset, which employs the Wilcoxon rank-sum test to pinpoint representative genes for each cluster. These genes helped ascertain the cellular identity of each cluster and served as markers for commonly occurring cell types in human bone marrow, as reported in the literature. Differentially expressed genes in each cell population were screened using the FindMarker function, with significance thresholds set at *p*_val_adj < 0.05 and avg_log2FC > 0.25. The selected genes underwent GO enrichment analysis using the cluster profiler package, followed by visualization. A detailed single-cell developmental trajectory was constructed using Monocle3. The original expression matrix was retrieved from the associated Seurat object via the GetAssayData function. After creating a CellDataset object in Monocle 3, we normalized and preprocessed the data using the preprocess_cds function. UMAP and dimensionality reduction functions were then employed to reduce the data’s dimensionality. Clustering and trajectory inference analysis were conducted using the cluster_cells and learn_graph functions, respectively. To comprehensively analyze immune cell–cell interactions, we utilized CellPhoneDB (v2.1.2), deriving potential ligand–receptor interactions based on receptor expression in one cell subset and ligand expression in another. Normalized counts from the total cell population and NK cell subgroups were used as inputs for the CellPhoneDB algorithm.

### 2.4 Clinical characteristics, immunophenotype, and treatment response of B-ALL patients

We conducted RNA-seq analysis of bone marrow samples from 139 newly diagnosed B-ALL pediatric patients aged 1–21, treated at Hunan Children’s Hospital from January 2020 to May 2023. Electronic medical records were analyzed for all patients. Eligibility criteria included a primary diagnosis of B-lineage ALL and completion of the entire standard chemotherapy regimen at the hospital. Diagnosis was established by bone marrow examination, based on morphologic and immunophenotypic findings. At diagnosis, we analyzed the proportion of leukemia blasts (immature cells) to the total lymphocyte count in the bone marrow smear of patients under the microscope. On the 19th day after treatment, the patient underwent minimal residual disease (MRD) evaluation with bone marrow puncture as a response to treatment.

### 2.5 Statistical analysis

All statistical analyses were conducted using R software (3.6.1) and GraphPad Prism 9. Data from different groups were compared using the Wilcoxon test and *t*-test. Unless otherwise stated, *p*-value < 0.05 was considered statistically significant.

## 3 Results

### 3.1 NK cells are reduced in proportion in peripheral blood and bone marrow at diagnosis

**Figure 1:**
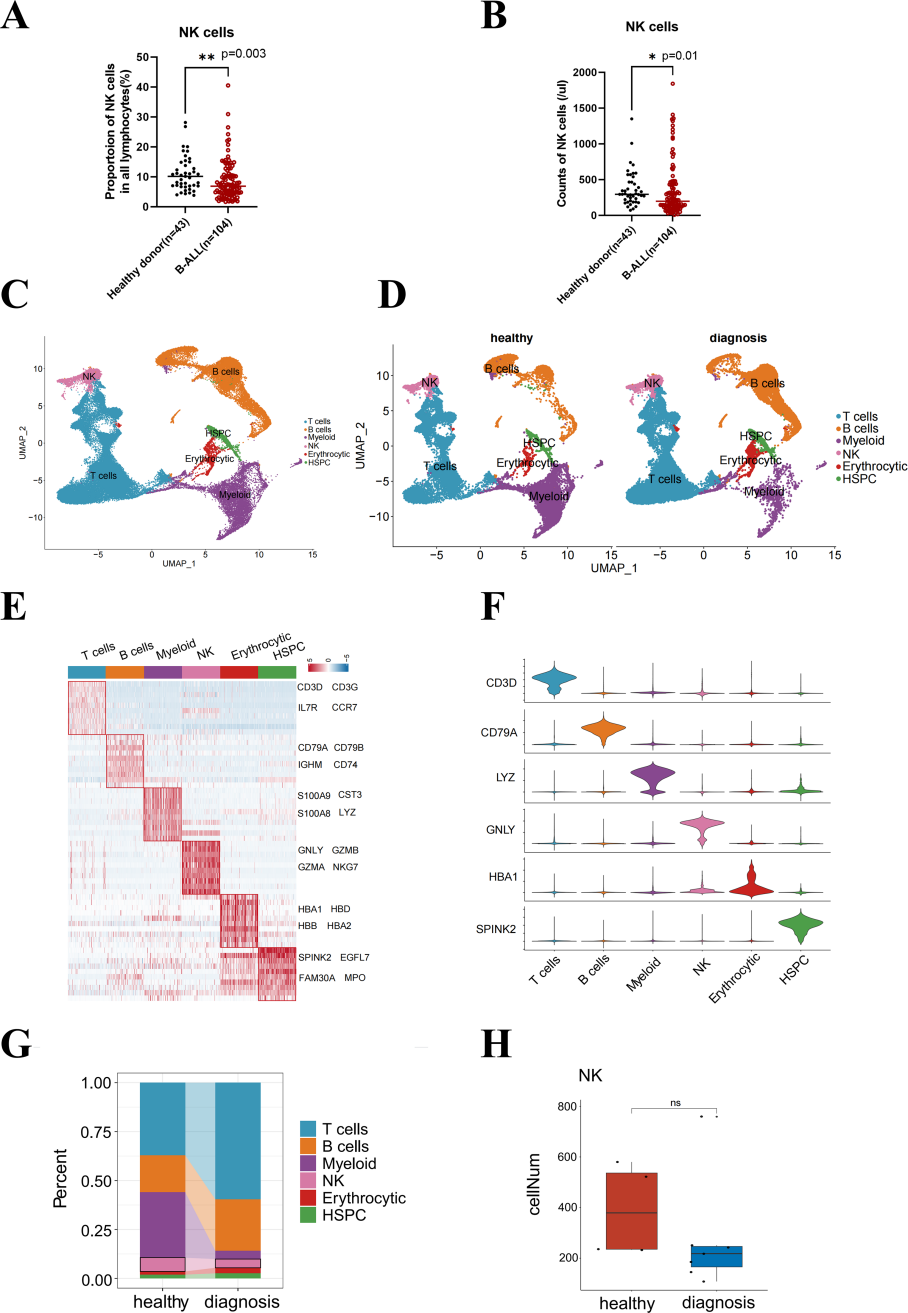
B-ALL remodels the distribution of lymphocyte subsets in healthy peripheral blood and bone marrow microenvironment. The counts **(A)** or proportion **(B)** of NK cell subsets in lymphocytes from healthy and pediatric B-ALL peripheral blood. **(C)** UMAP visualization of 65,397 individual cells from 11 primary bone marrow samples taken from healthy donors (*n* = 4), as well as B-ALL patients. **(D)** Marker-based cell type identification analysis allowed the prediction of six broad immune cell types across all profiled single cells in two groups. **(E)** Gene expression heatmap of the top 10 cell type-specific marker genes, as measured by the Wilcoxon rank-sum test (left), and the names of the top 4 marker genes are shown in the figure (right). **(F)** Violin plots showing the cluster-specific expression of the top-ranking novel candidate marker gene for each cell population identified by this study. **(G)** Bar plot showing the cell proportions of each immune cell cluster in the healthy and diagnosis (B-ALL) groups. **(H)** Box plot showing the cell numbers of individual samples in the healthy and diagnosis groups. Wilcox test was performed to measure differences in representation between healthy and diagnosis groups, with *p*-values indicated on the plots. *p < 0.05, **p < 0.01, “ns”, p > 0.05.

Using flow cytometry, we collected PBMC samples from 104 newly diagnosed ALL patients and 43 age-matched healthy donors and analyzed NK cell proportion and counts (per microliter of blood samples). The absolute counts and percentage of NK cells among PBMC were significantly lower in B-ALL patients compared to healthy controls (*p* = 0.003 and *p* = 0.016, respectively; **Figures 1A, B**).

The bone marrow produces immature cells that develop into leukemia cells (13, 14). Alterations in the immune microenvironment constitute a significant contributor to tumor immune evasion (15). Nevertheless, the influence of leukemia cells on the functional status, specifically the proliferation capacity, of NK cells in pediatric B-ALL remains unknown. We hypothesized that leukemia remodels the distribution of lymphocyte subsets in the B-ALL bone marrow microenvironment and inhibits the proliferation of NK cells. To address this, we downloaded single-cell sequencing data (GSE130116) from GEO for seven B-ALL patients at diagnosis and four healthy donors. We analyzed 65,397 transcriptomes from these samples to assess the composition of the immune bone marrow microenvironment composition (**Figure 1C**). We performed extensive unbiased clustering of all bone marrow cells into six broad hematopoietic cell types (**Figure 1D**), including T cells, B cells, NK cells, myeloid cells, and erythrocytic and hematopoietic stem and progenitor cells (HSPC), based on relative expression levels of manually curated gene signatures encompassing lineage-specific transcription factors, effectors genes, and surface markers (**Figures 1E, F**).

The emergence of bone marrow B-ALL elicits different responses from various hematopoietic lineages. We observed distinct reductions in the composition of the myeloid cell lineage, as well as a reduction in the fraction of NK cells at diagnosis compared to healthy bone marrow (**Figure 1G**). The cell numbers in the B-ALL groups declined compared with the healthy groups, although the difference was not statistically significant (**Figure 1H**). Therefore, we speculated that the proliferation or survival of NK cells was affected in the peripheral blood and bone marrow microenvironment of B-ALL.

### 3.2 Pronounced remodeling of the bone marrow microenvironment in B-ALL at diagnosis significantly alters NK subset proliferation

**Figure 2:**
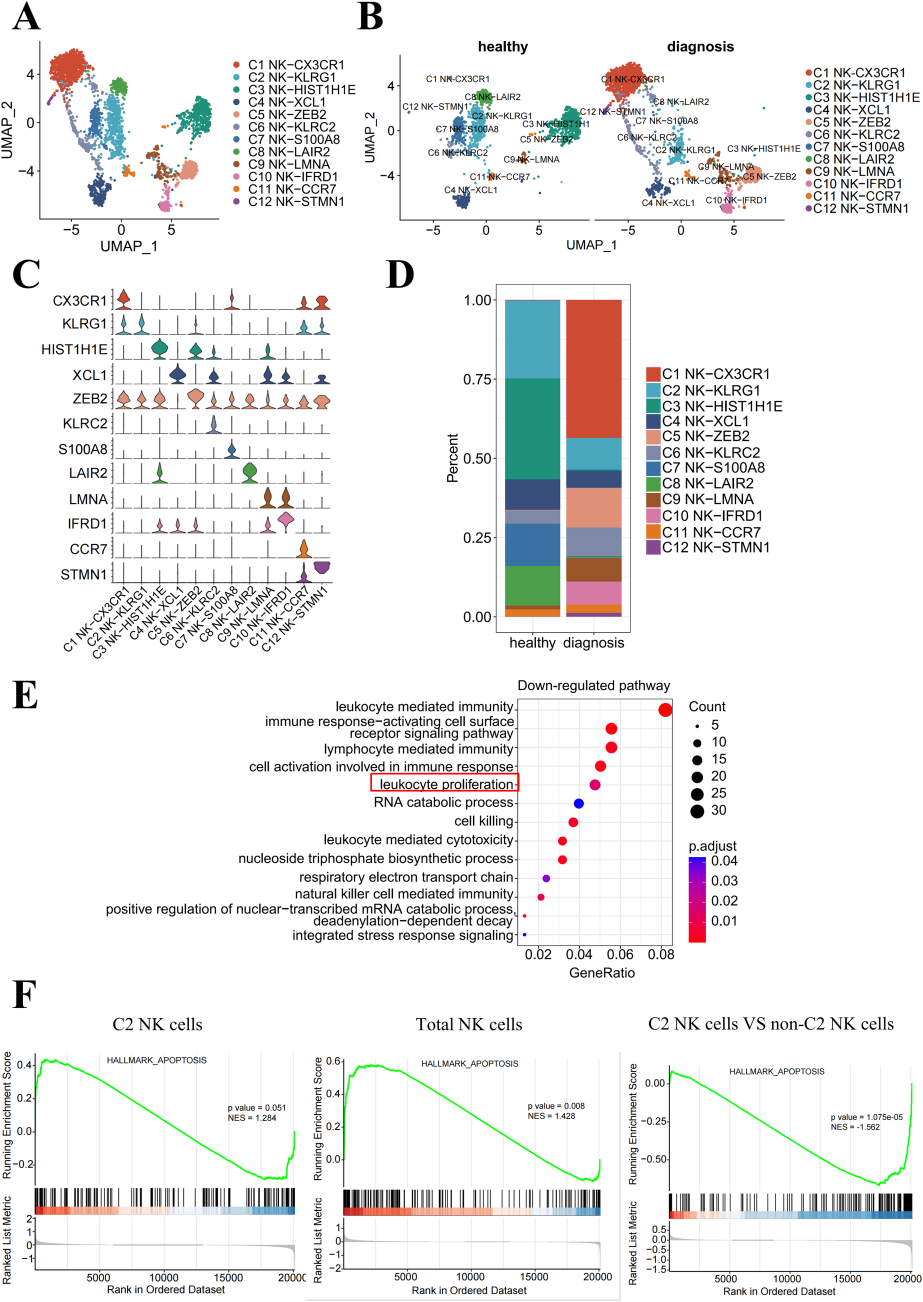
The proliferation of bone marrow-resident NK-KLRG1 cells is inhibited in the presence of leukemia. **(A)** UMAP visualization of NK cells from healthy donors and B-ALL patients, showing 12 transcriptionally distinct NK cell clusters overlaid on the UMAP representation. **(B)** UMAP plot of the NK cell subpopulations in the healthy and diagnosis groups. **(C)** Violin plots showing the cluster-specific expression of the top-ranking novel candidate marker gene for each cell population identified in this study. **(D)** Bar plot showing the cell proportions of each NK cell cluster in the healthy and diagnosis (B-ALL) groups. **(E)** Enrichment function analysis of differentially expressed genes in NK-KLRG1 (C2) cells between the diagnosis and healthy groups. The top 15 significantly downregulated (*p* < 0.05) pathways are shown. **(F)** GSEA plots reveal pathways enriched in C2 NK cells (left) and total NK cells (middle) derived from B-ALL patients compared with healthy volunteers, as well as C2 NK cells compared with non-C2 NK cells of B-ALL patients (right). NES, normalized enrichment score, calculated using the Kolmogorov–Smirnov test. The red box highlights a significant down regulation of the pathway in C2 NK cells (NK-KLRG1) between diagnosis and healthy groups in **(E)**.

The percentage of NK cells was significantly diminished because of the emergence of B-ALL in the bone marrow. To understand the specific NK subpopulations impacted by leukemia, we identified 12 transcriptionally distinct NK cell clusters (**Figures 2A, B**) based on the relative expression levels of signature gene markers (**Figure 2C**). Within the bone marrow, we observed a reduction in the fraction of NK cell clusters C2, C3, C4, C7, and C8 at diagnosis compared to the healthy groups (**Figure 2D**).

To validate which NK cell subset’s proliferative function has been significantly inhibited, we performed an enrichment analysis of validated human NK cell transcriptional signatures within our NK cell clusters and found that the “leukocyte proliferation” pathway was significantly downregulated in C2 NK cells (NK-KLRG1) between diagnosis and healthy groups (**Figure 2E**). In addition, the significantly downregulated (*p* < 0.05) pathways included leukocyte-mediated immunity, immune response-activating cell surface receptor signaling pathway, lymphocyte-mediated immunity, and cell activation involved in the immune response. This suggests that the function of C2 NK cells may also be impaired, providing more evidence for the potential dysfunction of C2 NK cells in B-ALL patients.

The Gene Set Enrichment Analysis (GSEA) revealed a significant enrichment of apoptosis-associated genes in C2 NK cells (*p* = 0.051, left) and total NK cells (*p* = 0.008, middle) in B-ALL groups compared to healthy groups within the bone marrow. Additionally, the analysis demonstrated a higher enrichment of these genes in C2 NK cells, as opposed to non-C2 NK cells, among B-ALL patients (*p* < 0.01, right) (**Figure 2F**). Collectively, these data indicate that the proliferation of C2 NK cells is suppressed, leading to enhanced cell apoptosis and subsequent dysfunction within the B-ALL microenvironment.

### 3.3 The association between the expression of KLF2 in B-ALL patients and clinical outcomes

**Figure 3:**
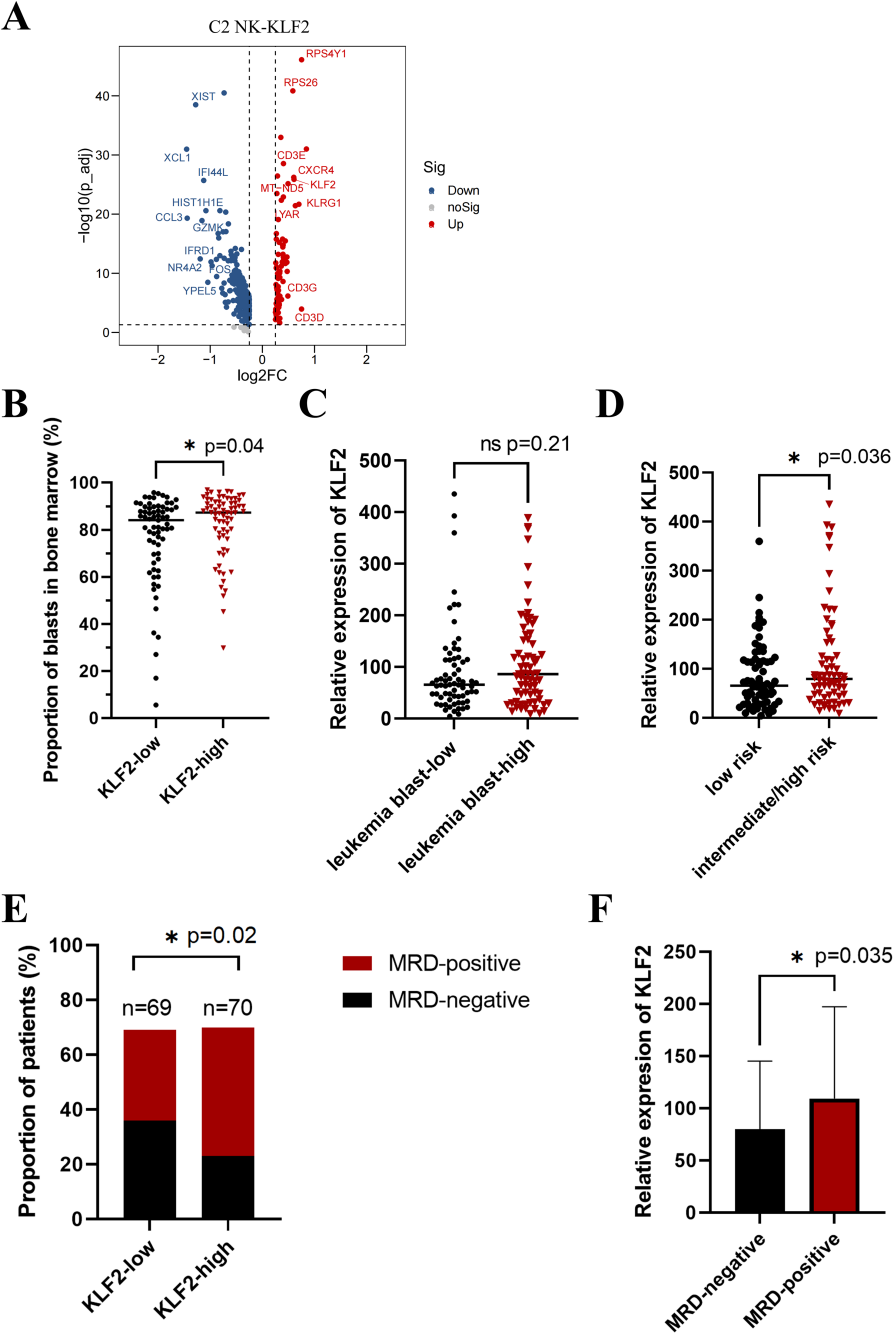
Overexpression of KLF2 in B-ALL predicts inferior clinical outcomes. **(A)** Volcano plot depicting the upregulated (right) and downregulated genes (left) in C2 NK-KLF2 cells compared with other NK cells in B-ALL patients. **(B)** The proportion of leukemia blast cells in the bone marrow in KLF2 low (*n* = 69) and KLF2 high (*n* = 70) groups of children with B-ALL at initial diagnosis. **(C)** The relative expression of the *KLF2* gene in the leukemia blast low (*n* = 69) and high (*n* = 70) groups in the bone marrow of children with B-ALL at initial diagnosis. **(D)** The relative expression of the *KLF2* gene in the bone marrow of children with B-ALL at diagnosis in the low-risk (*n* = 71) and intermediate/high-risk (*n* = 68) groups. **(E)** The proportion of MRD-positive and MRD-negative patients in the KLF2 low (*n* = 69) and high (*n* = 70) expression groups on the 19th day after standard treatment. MRD, minimal residual disease (MRD) refers to a state in which traces of leukemia cells remain in leukemia patients after receiving induction chemotherapy or bone marrow transplantation. **(F)** The relative expression of the *KLF2* gene in the bone marrow of children with B-ALL at diagnosis in the MRD-negative (*n* = 59) and MRD-positive (*n* = 80) groups. *p < 0.05, “ns”, p > 0.05.

Based on previous evidence that the proliferation of C2-NK cells is decreased, we focused on the specific signature genes of C2-NK cells to further investigate the key molecules that affect NK cell proliferation. To better examine the phenotype characteristics of decreased proliferation and survival in C2 NK cells in B-ALL patients, we further explored the expression of cytokines (*KLF2*, *TXNIP*, *S100A4*) and membrane proteins (*CD3E, CXCR4*) among the top 10 upregulated signature genes in C2-NK cells (**Figure 3A**). Previous studies have reported that the transcription factor KLF2 plays an important role in controlling NK cell expansion by inhibiting the proliferation of immature NK cells (CD27+CD11b−) in mice (16).

The role of KLF2 in B-ALL remains unclear. Is KLF2 a prognostic marker for B-ALL? To investigate this, we divided 139 newly diagnosed pediatric B-ALL patients into two equal groups: the KLF2-low and KLF2-high groups, based on their mRNA expression levels of KLF2, as determined by whole-gene transcriptome sequencing. Clinical characteristics are shown in **Table 1**. We evaluated the correlation between the percentage of leukemia blasts in bone marrow smears of newly diagnosed B-ALL patients and KLF2 expression. Strikingly, we found that in the KLF2-low group of B-ALL patients, the percentage of leukemia cells in the bone marrow was significantly lower than in the KLF2-high group at disease diagnosis (*p* = 0.04) (**Figure 3B**). This suggests that KLF2 may inhibit NK cell proliferation, promoting immune escape in leukemia and increasing the leukemic burden. Similarly, the leukemia blast-high group exhibited a modestly higher relative expression of KLF2 compared to the leukemia blast-low group, although the difference was not statistically significant (*p* = 0.21) (**Figure 3C**). Additionally, we compared the KLF2 expression levels in B-ALL patients with low-risk and intermediate/high-risk groups. The results show that the relative expression of KLF2 was higher in the intermediate/high-risk group (*p* = 0.036) (**Figure 3D**).

**Table 1:** Characteristics of B-ALL patients.

Characteristic	Patients of B-ALL (*n* = 139) No. (%)
Age at diagnosis (years, mean ± SD)	5.8 ± (1.3–16)
≤ 2 years	2 (1.4)
2–6 years	72 (51.8)
≥6 years	65 (46.8)
Sex	
Female	68 (48.9)
Male	71 (51.1)
Cytogenetic factors	
Normal	76 (54.7)
*BCR/ABL*-like fusions	9 (6.5)
*ETV6/RUNX1*	31 (22.3)
*IGH-DUX4*	1 (0.7)
*MLL/AF9*	6 (4.3)
*MLL/USP2*	1 (0.7)
*PHKB-CHD9*	1 (0.7)
*SENP6/PPIL4*	1 (0.7)
*TAF15/ZNF384*	1 (0.7)
*TCF3/HLF*	1 (0.7)
*TCF3/PBX1*	10 (7.2)
*TEL/AML1*	1 (0.7)
Chromosomal analysis	
Normal	108 (77.7)
Hypodiploidy	5 (3.6)
Hyperdiploidy	21 (15.1)
Unknown	5 (3.6)
Risk groups	
Standard (low) risk	71 (51.1)
Intermediate/high risk	68 (48.9)

Having demonstrated that KLF2 is a risk factor influencing the progression of B-ALL, we would like to know whether KLF2 can predict the prognosis of B-ALL patients. Monitoring of MRD is crucial for accurately predicting the prognosis of B-ALL. Patients with positive MRD (MRD-positive) tend to have a poorer prognosis compared to those with MRD-negative. We evaluated the MRD results of 139 B-ALL patients on the 19th day after standard chemotherapy. We found that the positive rate of MRD was significantly higher in the KLF2-high group than in the KLF2-low group (*p* = 0.02) (**Figure 3E**). Likewise, the relative expression of KLF2 was higher in the MRD-positive group (*p* = 0.04) (**Figure 3F**). These findings, in combination with our scRNA-seq data from the bone marrow of B-ALL patients, suggest that transcription factor-KLF2 decreases the proliferation of NK cells and impairs their ability to eliminate B-ALL. The KLF2 expression may be associated with inferior treatment outcomes.

### 3.4 B cells play an important role in the development of NK cells in B-ALL

**Figure 4:**
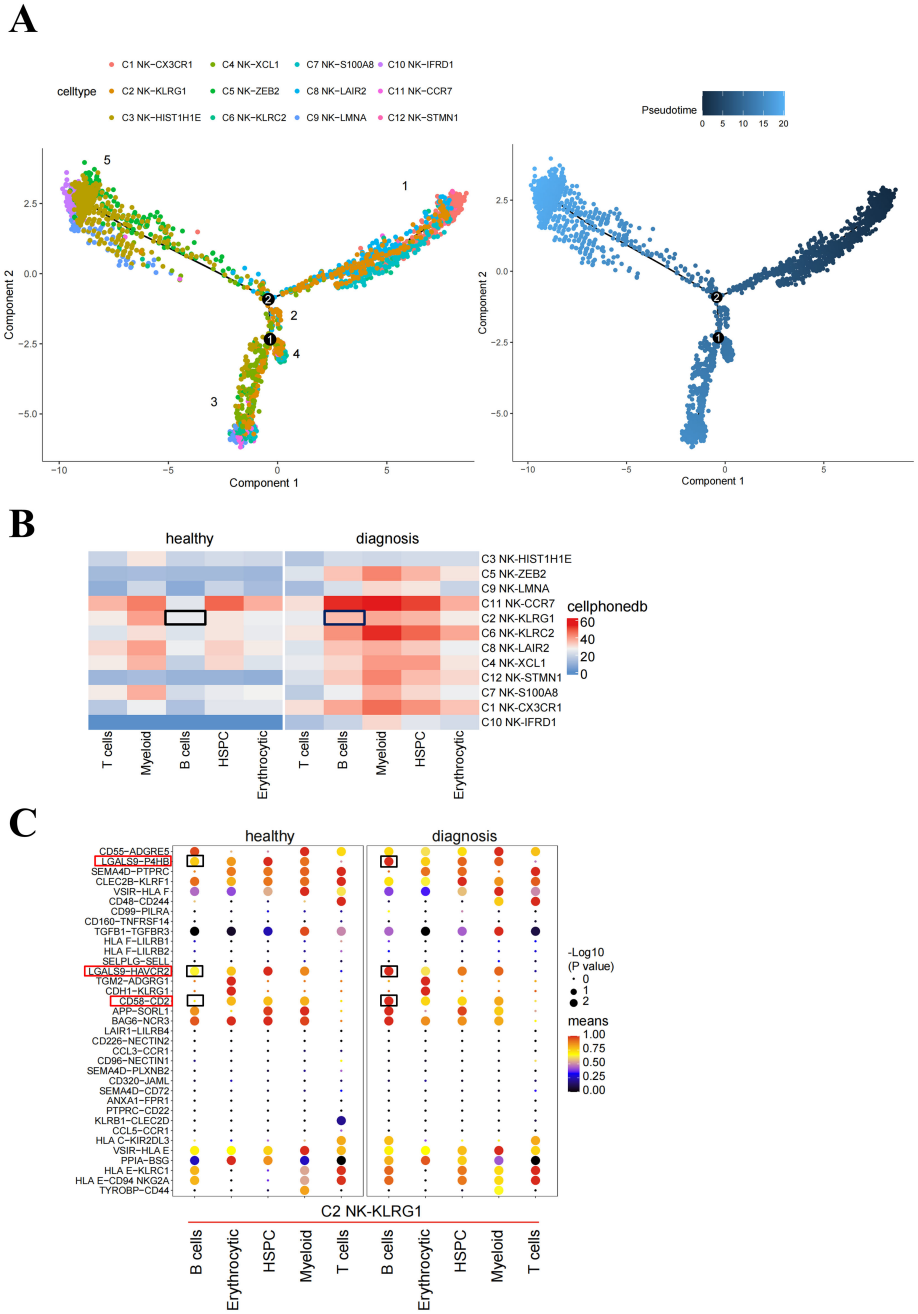
Potential pathways affecting NK cell proliferation. **(A)** Pseudotime trajectory analysis of NK cell subsets. Each dot represents a single cell, color-coded according to its cluster label. The integrated diffusion maps of NK cell clusters reveal a developmental trajectory with conserved “pseudo-temporal” dynamics. **(B)** Heatmap of the number of significant cell–cell interactions identified between NK cell subsets as senders and immune cell subsets as receivers. **(C)** Comparison of significant ligand–receptor pairs between immune cell subsets and NK-KLRG1 cells, highlighting the ligand–receptor interactions between B cells and NK-KLRG1 cells. Dot color reflects communication probabilities, and dot size represents computed *p*-values. Space indicates that the communication probability is zero. *p*-values are computed from the one-sided permutation test. The black box indicates the immune cell subsets that interact with C2 NK cells between healthy and diagnosis groups in **(B)**. The red and black boxes represent immune checkpoint-related receptor ligand pairs with significantly enhanced interaction between B cell-C2 NK cells in diagnosis compared healthy groups in **(C)**.

To investigate how NK cells develop in B-ALL, we performed trajectory analysis using 12 NK clusters. The integrated diffusion maps of NK clusters revealed a developmental trajectory with “pseudo-temporal” dynamics. The differentiation of C2 NK cells was shown to occur throughout the entire developmental trajectory, spanning from status 1 to 4 (**Figure 4A**), indicating that KLRG1 may play an important role in the development of diseases.

To compare the differences in ligand–receptor interactions between different hematopoietic cells and C2 NK cells in healthy patients and B-ALL patients, we performed cell–cell interaction analyses. The results from CellPhoneDB indicated that healthy donors and B-ALL patients exhibited different cell interactions. Specifically, B cells and C2 NK cells exhibited higher outgoing interaction strength in B-ALL patients compared with healthy donors (**Figure 4B**). We speculated that the B cells in the bone marrow of B-ALL patients include the majority of leukemia cells, which inhibit the proliferation of C2 NK cells. Consequently, we investigated the signaling pathways through which B cells act as senders and C2 NK cells as receivers, in subsequent analyses. From healthy donors to B-ALL patients, the signaling network and related ligand–receptor interactions, including LGALS9-P4HB, LGAL9-HAVCR2, and CD58-CD2, showed significantly higher communication probability in B cell–C2 NK cell interaction in B-ALL (**Figure 4C**). In summary, these data implicated that B cells may act on NK cells, promoting B-ALL progression *in vivo*.

## 4 Discussions

NK cells are an important component of the innate immune system and show promise in patients with a variety of malignancies (1). NK cells have been utilized for the treatment of cancer in patients with varying success, including mismatch of NK inhibitory receptor and MHC ligand interactions in the context of HCT, NK cell adoptive immunotherapy, and administration of antibodies, cytokines, or drugs aimed at enhancing NK cell function (7–9). If immunotherapy for childhood ALL is to prove successful, a better understanding of the impact of leukemia on the NK cells is critical (17–19). However, NK cell-based immunotherapy has been limited due to small numbers, short lifespan, exhaustion, inhibitory receptor-induced inhibition, poor trafficking, and poor tumor infiltration (1, 10). Therefore, it is urgent to investigate the impact of leukemia on the proliferation and survival of NK cells and to develop novel strategies for enhancing NK cell counts and extending their effector functions *in vivo*.

In this study, we report that the proportion and numbers of NK cells were significantly decreased in pediatric B-ALL patients compared to healthy donors in peripheral blood. Furthermore, we analyzed published scRNA-seq data from pediatric B-ALL and normal bone marrow samples and showed that the proliferation of NK cells was decreased in pediatric B-ALL. We demonstrated that the “leukocyte proliferation” pathway was significantly downregulated in C2 NK cells in B-ALL patients at diagnosis and that C2 NK cells overexpress KLF2. Prior work has documented that KLF2 limits antigen-independent NK cell proliferation in all tissues of mice, and removal of this factor expands the proliferative burst associated with CD27+CD11b− NK cells (16). In addition, we found a significant enrichment of apoptosis-associated genes in C2 NK cells overexpressing KLF2. These findings are consistent with previous reports (16). Finally, we validated our findings through the analysis of bulk transcriptomic and clinical data from a cohort of 139 B-ALL patient bone marrow samples. We found that the expression of KLF2 in B-ALL at diagnosis was positively correlated with the percentage of leukemia cells and the positive rate of MRD, indicating that KLF2 is a marker of poor prognosis. This study therefore suggests that KLF2 decreases the proliferation of NK cells and impairs their ability to eliminate B-ALL.

Most notably, this is the first study, to our knowledge, to investigate the effect of KLF2 on NK cells in B-ALL children. Our results provide a therapeutic means of targeting KLF2 to promote the expansion and long-term survival of NK cells and improve NK cell engraftment and sustainability in cancer patients. However, some limitations are worth noting. This raises the question of what factors directly promote KLF2 transcription in NK cells and what pathways through which KLF2 inhibits NK cell proliferation. Future work should therefore include mechanistic research to determine the role of KLF2 in inhibiting NK cell proliferation, as well as follow-up work designed to identify strategies for promoting NK cell expansion and survival.

## Data Availability

The original contributions presented in the study are publicly available. This data can be found here: https://dataview.ncbi.nlm.nih.gov/object/PRJNA1208733?reviewer=4qg0lfl1d0i0l1esd7bb6n4s88 number: PRJNA1208733. Also, publicly available datasets were analyzed in this study. This data can be found here: https://www.ncbi.nlm.nih.gov/geo/query/acc.cgi?acc=GSE130116/accession number GSE130116.
